# From Nose Job to a Better Job: A Scoping Review of Facial Aesthetics, Attractiveness Bias, and Outcomes in the Workplace

**DOI:** 10.1093/asjof/ojag104

**Published:** 2026-06-08

**Authors:** Justin L Anderson, Joey Liang, Jackson M Cathey, Jessica Berns, Samantha J Kaplan, Ashit Patel

## Abstract

Facial appearance strongly influences professional and social outcomes, with individuals perceived as more attractive often receiving increased job opportunities—a phenomenon known as “attractiveness bias.” As facial plastic surgery becomes increasingly common, its potential impact on workplace dynamics and ethics warrants evaluation. This scoping review synthesizes current evidence examining the relationship between facial aesthetic surgery, attractiveness bias, psychosocial outcomes, and professional trajectories. A comprehensive search of the MEDLINE database via PubMed, Cochrane Library, SCOPUS, and PsycINFO was conducted (inception-September 10, 2025) according to PRISMA-ScR guidelines. Controlled vocabulary (MeSH) and free-text terms related to facial aesthetic surgery, career outcomes, and attractiveness bias were combined. Studies were included if they examined surgical facial aesthetic or reconstructive procedures and assessed self-perception, attractiveness bias, professional or socioeconomic outcomes, or appearance-related psychological distress. Sixty-one studies met inclusion criteria and were synthesized qualitatively into 3 conceptual domains. Across studies, facial aesthetic surgery consistently enhanced both self-perception and external evaluations. Postoperative images were rated as more attractive, trustworthy, competent, and intelligent than preoperative images. Patients reported improved self-esteem and social functioning, with several studies identifying career advancement as a key motivator. In contrast, individuals with facial trauma, congenital malformations, or features diverging from cultural beauty norms reported discrimination and lower levels of overall satisfaction. Rates of body dysmorphic disorder among surgical candidates ranged from 2.5% to 32%. Facial aesthetic surgery may influence professional outcomes by modifying self-confidence and how others perceive competence. Plastic surgeons should recognize workplace-related motivations during consultation and incorporate ethical counseling to ensure informed decision-making. Further longitudinal research is needed to clarify whether cosmetic enhancement leads to tangible increased earnings.

**Level of Evidence:** 3 (Therapeutic) 
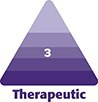

Physical appearance influences social and professional interactions. Research consistently links facial aesthetics with favorable social and workplace outcomes, with individuals perceived as more attractive often receiving higher salaries, better job opportunities, and faster promotions.^1,2^ This phenomenon, commonly referred to as “attractiveness bias” or “pretty privilege,” suggests that facial appearance influences not only personal confidence but also how others evaluate competence, intelligence, and social skills.^3-5^

Among cosmetic procedures, rhinoplasty, blepharoplasty, and facelifts particularly have been linked to enhanced self-perception and improved external ratings.^6,7^ Patients undergoing these procedures frequently report increased confidence, which may translate into greater assertiveness and engagement in professional environments.^8,9^ Furthermore, studies have shown that observers rate individuals postaesthetic surgery as more competent, trustworthy, and capable, reinforcing the notion that facial aesthetics plays a tangible role in career-related opportunities.^10^

Conversely, individuals who are viewed as less attractive or those with craniofacial abnormalities or facial features diverging from the canonical views of attractiveness have reported experiencing discrimination in employment and social settings, highlighting the extent to which facial appearance influences professional interactions.^11^ This raises 3 ethical considerations: (1) to what extent does facial plastic surgery serve as a tool for career advancement? (2) does facial plastic surgery reinforce workplace inequalities based on appearance, or does it allow for a level playing field? (3) to what extent do these pressures affect the mental health of potential facial plastic surgery patients?

As the demand for cosmetic surgery continues to rise, particularly among professionals seeking to maintain a competitive edge, examining the relationship between facial plastic surgery, career outcomes, and the broader societal implications of attractiveness bias in professional settings is essential. By proactively addressing this motivation during preoperative counseling, plastic surgeons can better manage expectations, support ethical decision-making, and contribute to more durable postoperative satisfaction.

## METHODS

### Search Strategy

A comprehensive search of the following databases was conducted by a medical librarian with expertise in systematic searching (S.J.K) in accordance with PRISMA-ScR guidelines: MEDLINE via PubMed, Cochrane Library, SCOPUS, and PsycINFO. The search combined controlled vocabulary (MeSH) and free-text terms related to facial aesthetic surgery, career outcomes, and perceived attractiveness or bias. The complete search string is provided in Appendix 1. Dates include from inception to September 10, 2025. Only studies in English were included.

#### Study Selection

All identified records were imported into Covidence systematic review software (Veritas Health Innovation, Melbourne, Australia). Two independent reviewers (J.L.A. and J.M.C.) screened all titles and abstracts for relevance. Studies were eligible for inclusion if they:

1. Examined surgical facial aesthetic or reconstructive procedures (eg, rhinoplasty, blepharoplasty, rhytidectomy, or orthognathic reconstruction)
2. Assessed self-perception, attractiveness bias, or professional and socioeconomic outcomes (eg, employability, income, promotion, workplace discrimination, or perceived competence)
3. Investigated mental health or psychosocial outcomes associated with facial appearance, both positive (ie, postsurgical) or negative (eg, distress, anxiety, depression, or reduced quality of life linked to malformation or perceived unattractiveness [self-reported or externally perceived]).

Studies that focused solely on nonfacial or dental procedures lacked a clear methodological framework—including narrative discussions published in nonmedical literature—and case reports or letters to the editor were excluded. Inter-rater reliability during title/abstract and full-text screening was assessed using Cohen's kappa statistic automatically generated within Covidence, interpreted according to standard guidelines: <0 indicating poor agreement, 0.00 to 0.20 as slight, 0.21 to 0.40 as fair, 0.41 to 0.60 as moderate, 0.61 to 0.80 as substantial, and 0.81 to 1.00 as almost perfect agreement.^12^ Discrepancies during screening were resolved through discussion and consensus between reviewers.

#### Data Extraction

Data from included studies were independently extracted by 2 reviewers using a standardized form that captured study design, population characteristics, intervention or exposure, outcome measures, and key findings. All statistical values (eg, *P*-values, effect sizes, and odds ratios) reported in this review were extracted directly from the original source studies; no independent statistical analyses were performed by the authors. Extracted data were iteratively reviewed to identify recurring patterns across studies. Disagreements were resolved through discussion until consensus was achieved.

#### Data Synthesis and Thematic Analysis

Given substantial methodological heterogeneity identified during full-text review, a qualitative synthesis was undertaken to characterize patterns across the literature, as a quantitative meta-analysis was determined to be inappropriate. Conceptual domains were developed inductively during the review process after substantial heterogeneity in methodologies and reported outcomes became apparent. Thematic categorization was performed independently by 2 reviewers (J.L.A. and J.M.C.), with discrepancies resolved through discussion.

## RESULTS

**Figure 1.**
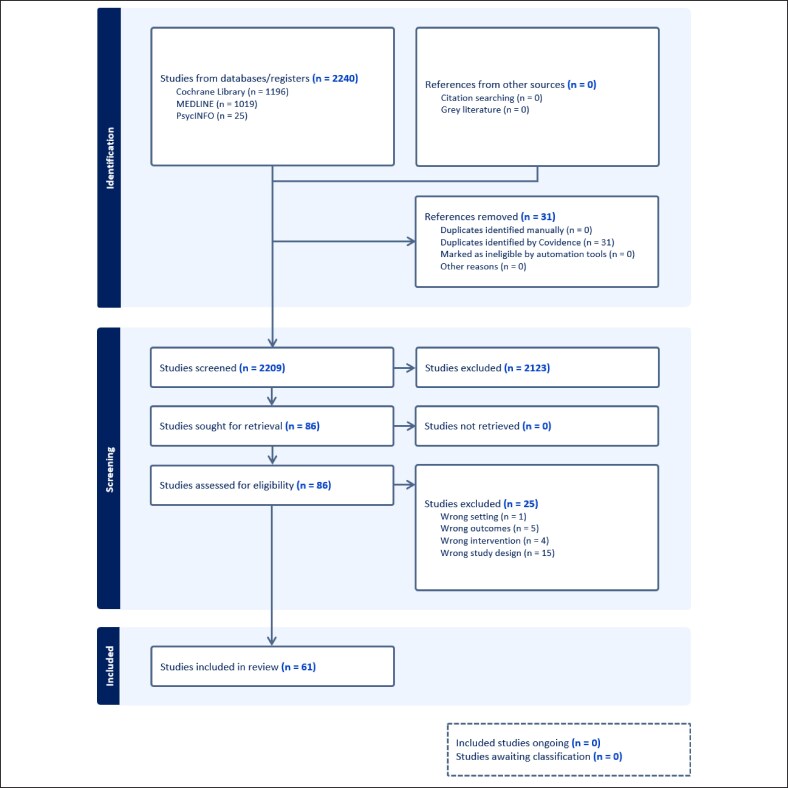
PRISMA flow diagram of study selection. PRISMA flow diagram illustrating the identification, screening, eligibility assessment, and inclusion of studies examining the relationship between facial aesthetic surgery, attractiveness bias, psychosocial outcomes, and professional trajectories.

A total of 2240 records were identified across all databases. Screening and data extraction were performed using Covidence, which removed 31 duplicates. After title and abstract screening, 86 studies were reviewed in full text, with 61 meeting inclusion criteria (Figure 1, Supplemental Table 1). Inter-rater reliability demonstrated almost perfect agreement during title and abstract screening (Cohen's κ = 0.862) and full-text review (κ = 0.850).

A quantitative meta-analysis was not performed due to substantial methodological heterogeneity across included studies. Study designs ranged from experimental image-rating paradigms to observational surveys and qualitative interviews; populations included patients, clinicians, and lay observers; and outcomes varied widely, encompassing attractiveness ratings, psychosocial instruments (eg, FACE-Q), employment status, and perceived professional competence. Additionally, effect sizes and variance estimates were inconsistently reported, precluding meaningful statistical pooling. Due to this variability, subgroup meta-analyses were not feasible without introducing substantial risk of misinterpretation. Therefore, findings were synthesized narratively within 3 conceptual domains that emerged from the literature: External perception and attractiveness bias (19 studies), self-perception and psychosocial outcomes (29 studies), and mental health and appearance-related distress (13 studies).

### External Perception and Attractiveness Bias

Studies in this domain examined how facial plastic surgery or aesthetic interventions influence others’ perceptions of attractiveness, competence, trustworthiness, or professional capability.^6,10,13-29^ Across experimental and survey-based studies, postoperative photographs were viewed more favorably than subjects’ preprocedure photos; this was evaluated through the use of questionnaires after viewing both preprocedure and postprocedure photos in many studies. While lower perceived age or increased attractiveness scores were consistent and would be reasonably expected, subjects were also viewed as more confident, trustworthy, healthy, successful, and intelligent.^6,10,13,15,18,21-23,25,29^ Patients with facial deformities or with noses that are not aligned with the societal perception of beauty received worse personality rankings—including being perceived as less honest (*P* = .007), employable (*P* = .001), capable (*P* = .002), and intelligent (*P* = .03)—and reported more stigmatizing behaviors across age demographics, ranging from higher risk of experiencing teasing or confused staring as a child (d = 0.81, *P* = .003) to appreciable gaze deviation to defect in adult patients (ƛ=0.9568, F = 17.26, *P* < .0001), further strengthening the separation between those that have received surgical intervention and those who have not.^20,24,27,29^ Conversely, Abousy et al^13^ demonstrated that this effect is correctable, noting that patients were perceived as globally more professionally competent and socially accepted following facial transplantation (*P* < .001). In healthcare, patients expect to experience less pain from “a painful procedure” (B = −0.23, t = −10.59, *P* < .001) and are more likely to use over-the-counter pain medication vs prescription pain medication (B = −0.18, t = −6.39, *P* < .001) based solely on appearance of their provider.^26^

### Self-Perception and Psychosocial Outcomes

This domain encompassed studies focused on patients’ internal experiences, motivations, and self-assessment following facial plastic surgery.^9,30-57^ Reported outcomes included self-esteem, body image, confidence, and quality of life. Increased self-esteem and confidence were consistently identified as primary motivations for pursuing facial plastic surgery.^30,36,37,40,41,43,46,47^ The effects of media—particularly social media—had mixed results on patients but trended toward negative effects on self-esteem scores and increased desire for cosmetic surgery, including the top experienced sociocultural pressure in one group, as determined by the Sociocultural Attitudes Towards Appearance Questionnaire-4-Revised (2.28, SD 1.31, *P* < .001).^32,49-51^ One study showed increased social media addiction scores (2.083, *P* = .04) among rhinoplasty patients, but no differences in meeting diagnostic threshold nor differences in self-esteem (−0.377, *P* = .707) or body perception (1.387, *P* = .168).^44^

Increased confidence postoperatively was seen, including an increase of 2.33 ± 1.03 (*P* < .001) on the Rhinoplasty Outcome Evaluation^9^ and an increase of 12.95 points (71.23 vs 58.28, *P* < .001) on the FACE-Q outcome measure.^39^ Similar increases in self-esteem postoperatively were appreciated across studies, including an increase in the Rosenburg self-esteem score of 0.12 (±0.31, *P* < .001)^9^ or a score postoperatively that was above that of the general population (mean=35.4, *P* < .001).^45^ Increases in body image were also appreciated postoperatively, with an increased score of 0.94 (±0.82, *P* < .001)^48^ on the Rhinoplasty Outcome Evaluation and a change of −0.163 (± 0.177, *P* = .01) toward the patient's ideal self of the Repertory Grid.^53^

“Improved eating” was the main perceived benefit identified preoperatively in 24% of orthognathic surgery patients (15/64); however, only 13% (8/64) of patients stated this was the main benefit postoperatively; instead, “improved facial appearance” was identified as the greatest benefit postoperatively in 21% of patients (13/64).^38^ Additionally, 7 studies state that one of the main motivations for undergoing surgery is career advancement or confidence in the workplace, ranging from 28.52% (414/1452) to 37.2% (1206/3228) of patients’ identified motivations.^30,31,36,37,45,50,52^ In one study, patients noted an improvement in “work” postsurgery (*t* = 2.503, *P* = .02) as determined by the Questions on Life Satisfaction Modules (FLZ^M^) assessment tool.^45^ These trends transcend cultural boundaries, with high rates of rhinoplasty in the Middle East and blepharoplasty in East Asia—where 1 of 6 independent predictors of obtaining the surgery was a desire for socioprofessional advancement, reflecting globalized aesthetic norms toward westernization or “Europeanization” of facial aesthetics.^31-33,35,37,41^ Notably, psychosocial outcomes were not uniformly positive across all literature; for example, Viana et al^55^ reported worsened self-esteem in 14% (7/50) of patients following lower blepharoplasty, though all affected patients experienced major concurrent life stressors (eg, divorce, conflict, and bereavement). In contrast, patients who had experienced facial trauma reported higher rates of unemployment (χ^2^  *P* = .01) and lower satisfaction with their lives (21.2, SD 7.7 vs 27.8, SD 5.2).^42^

### Mental Health and Appearance-Related Distress

Studies categorized under this domain explored the relationship between facial appearance, mental health conditions, and psychological distress.^58-69^ Topics included the prevalence of depression, anxiety, and psychiatric conditions, including body dysmorphic disorder (BDD), among patients seeking or having undergone facial plastic surgery. A common theme was struggling to fit in to societal expectations of beauty, leading to increased anxiety in social situations; this effect was noted for males and females.^58,60,62,64,67^ Rates of body dysmorphic disorder amongst patients seeking surgery ranged from 2.5% to 32% across identified studies.^63,66,67,70^ About 93.1% (23/29) of adult patients with cleft lip and palate reported difficulties in social relationships due to their appearance and negative self-image, making them more likely to screen positive for body dysmorphic disorder (OR 10.5, CI 2.7-41.1).^67^ Other psychiatric pathology, including the presence of a personality disorder or having a depressive-type adjustment disorder showed prevalence (40%, 10/25), with the latter showing an improvement in symptoms postfacial cosmetic surgery (50%, 3/6).^68^ Having higher levels of neuroticism or psychoticism as personality traits was found to be associated with higher rates of facial plastic surgery, specifically for rhinoplasty (OR 1.07 for neuroticism) and blepharoplasty (OR 1.04 for neuroticism, OR 1.10 for psychoticism).^69^ Being assigned to a certain group—including racial or age categories—was also identified as a source of stress, resulting in experienced discrimination across domains. Age was identified as the main overall reason for experienced discrimination (31.9%, 16/50) in interpersonal (36.0%), work (20%), and healthcare (16.0%) settings.^65^ Being perceived as a “Afro-Venezuelan” led to a much higher desire to have a rhinoplasty (50% of interviewed patients) vs those perceived as “blanca” (16%) or “morena” (16%) due to perceived social discrimination across settings.^60^

## DISCUSSION

This review indicates that facial aesthetics may play a more significant role in professional outcomes than previously recognized. While attractiveness bias has long been documented in hiring and promotions, this review highlights the potential for facial cosmetic surgery to influence workplace interactions by altering self-perception and external perception simultaneously. Notably, this effect appears to be cross-cultural, reinforcing the idea that certain aesthetic procedures are not just about individual confidence but also about aligning with broader social and professional expectations.^31-33,35,37,41^ Despite these generally positive trends, the psychosocial outcomes of surgery were not uniformly favorable. As noted previously, while some patients reported worsened self-esteem postoperatively, this was largely attributed to major concurrent life stressors rather than the surgical outcome itself. All patients—including those with worse Rosenberg scores—did report positive changes in their social lives and relationships, suggesting that postoperative psychological trajectories are shaped by broader contextual factors rather than surgical outcomes alone.^55^

Additionally, high variability was seen in rates of BDD across identified studies. Upon review of these articles, different methods of assessing BDD were utilized, including the Body Perception Scale,^67^ the Body Dysmorphic Disorder-Yale Brown Obsessive Compulsive Scale,^67^ and the Cosmetic Procedure Screening Questionnaire.^66^ In addition, these studies were generally conducted in one clinic or practice, limiting the generalizability to the population at large. This heterogeneity likely led to the differences in reported rates of BDD seen in this review.

A consistent finding is the contrast between individuals who undergo facial cosmetic surgery and those with craniofacial abnormalities or asymmetries. While cosmetic surgery patients are often perceived as more competent and socially adept, individuals with facial differences frequently report discrimination in hiring and workplace settings. These differences include not just having an anatomical anomaly, such as those noted in congenital malformations or secondary to trauma but also includes being perceived as older—as discussed by Pearl et al^65^—or belonging to a minority ethnic group—as discussed by Gulbas.^60^ This disparity is reinforced by studies in which participants consistently assign lower personality ratings to individuals based solely on their appearance. In addition, Levine et al^42^ showed that rates of unemployment increased after experiencing facial trauma and were higher than the control group (*P* = .01), further supporting the stark contrast in professional opportunities based on facial appearance. This effect appears to be mitigated through reconstructive efforts; as previously highlighted, procedures such as facial transplantation have been shown to restore perceived professional competence and social acceptance.^13^ These results underscore the deeply ingrained nature of appearance-based biases in both social and professional landscapes.

The findings of this review provide compelling evidence that facial aesthetic procedures influence both self-perception and external evaluations, with implications for professional settings, suggesting that facial cosmetic surgery may be leveraged—consciously or unconsciously—as a tool for professional advancement. These findings carry important implications for surgical decision-making. First, should plastic surgeons consider career motivations when evaluating candidates for surgery? Second, is it ethical to promote surgical enhancement as a means of professional advancement?

While correction of gross deformities may level the professional playing field, cosmetic enhancement of normal facial appearance may be perceived as a mechanism for professional progression. While the literature consistently demonstrates improvements in perceived competence and social evaluation, direct evidence linking facial aesthetic surgery to objective career outcomes remains limited. The current body of research therefore supports a conceptual, rather than causal, relationship between aesthetic intervention and professional advancement. Additionally, there are certainly moderators and mediators to this pathway, including baseline attractiveness, gender, race, socioeconomic status, occupational field, and organizational culture. However, this thought is already in the minds of patients, as seen across studies identified in this review.^30,31,36,37,45,50,52^ If certain procedures become de facto prerequisites for success in high-visibility fields, it may place undue pressure on individuals—particularly women and minorities, the latter of whom have reported less satisfaction postfacial cosmetic procedure—to conform to specific aesthetic norms to remain competitive. While patient autonomy remains central to medical ethics, surgeons should refine their preoperative counseling strategies to ensure that individuals are making informed decisions free from external pressures or unrealistic expectations. This can be added to the current framework for how patients are screened for body dysmorphic disorder or other psychological pathology, which may affect patient satisfaction with the outcome of surgery.

### Limitations and Future Directions

While this review synthesizes current literature on attractiveness bias, facial plastic surgery, and workplace interactions, it also highlights gaps that future research must address, including a lack of longitudinal data. Based on this review, no studies were identified that track career outcomes (eg, promotions, salary changes) before and after surgery. While there is some extrapolated evidence of this across different disciplines, there is a paucity of literature describing how many individuals explicitly undergo surgery for workplace benefits or, more importantly, if they obtain their desired benefits. Rather, this work is intended to bring about important questions regarding the topic of leveraging facial aesthetic procedures for personal career gain and explores ethical and societal implications and the lack of clear data in the literature. Future investigations should clarify whether facial cosmetic surgery perpetuates appearance-based disparities or promotes equity in professional settings by integrating patient-reported motivations with longitudinal data on income and career progression.

## CONCLUSIONS

The intersection of facial aesthetics, workplace bias, and career success represents an evolving and ethically complex field. While cosmetic surgery may enhance personal confidence and professional engagement, it also raises critical questions about fairness, implicit bias, and social pressures in the workplace. Future research should explore whether societal trends are shifting toward the professionalization of facial aesthetics—and what role plastic surgeons should play in that discussion. While improved confidence and self-perceptions are valid motivations, surgeons should be mindful of patients seeking surgery primarily for professional advantage, engaging in nuanced preoperative counseling to ensure realistic expectations in order to optimize patient outcomes.

## Supplemental Material

This article contains supplemental material located online at https://doi.org/10.1093/asjof/ojag104.

## Supplementary Material

ojag104_Supplementary_Data
