# Supplementary material for: From Nose Job to a Better Job: A Scoping Review of Facial Aesthetics, Attractiveness Bias, and Outcomes in the Workplace
Source: Aesthet Surg J Open Forum. 2026 Jun 8;8:ojag104. doi: 10.1093/asjof/ojag104 (PMC13322391; doi:10.1093/asjof/ojag104)
Supplement: ojag104_Supplementary_Data [file ojag104_supplementary_data.zip › NJtBJ Supplementary Appendix 1 .docx]

**Search Strategy Report: Original Search**

Topic: **1) to what extent does facial plastic surgery serve as a tool for career advancement? and 2) does facial plastic surgery reinforce workplace inequalities based on appearance, or does it allow for a level playing field?**

Searcher: SJK

Date: 9.10.2025

Database (including vendor/platform): MEDLINE (via PubMed)

| **Set #** | **Search Strategy** | **Results** |
| --- | --- | --- |
| **1. Facial Plastic Procedures** | **"Rhinoplasty"[Mesh] OR "Blepharoplasty"[Mesh] OR "Rhytidoplasty"[Mesh] OR ((cosmetic[tiab] OR aesthetic[tiab] OR facial[tiab]) AND (surger*[tiab] OR procedur*[tiab] OR operati*[tiab])) OR facelift[tiab] OR facelifts[tiab] OR “jawline contouring”[tiab] OR rhinoplasty[tiab] OR rhinoplasties[tiab] OR blepharoplasty[tiab] OR blepharoplasties[tiab] OR Botox[tiab] OR ((face[tiab] OR facial[tiab] OR lip[tiab] OR lips[tiab] OR cheek[tiab] OR cheeks[tiab]) AND (inject*[tiab] OR filler[tiab] OR fillers[tiab])) OR rhytidoplasty[tiab] OR rhytidoplasties[tiab] OR “face lift”[tiab] OR “face lifts”[tiab] OR septorhinoplast*[tiab]** | **110432** |
| **2. Bias** | **"Employment"[Mesh] OR "Job Application"[Mesh] OR “Personnel Selection”[mesh] OR career*[tiab] OR workplace*[tiab] OR job[tiab] OR jobs[tiab] OR hired[tiab] OR hiring[tiab] OR hire[tiab] OR hires[tiab] OR employ*[tiab] OR unemploy*[tiab] OR work*[tiab] OR interview*[tiab] OR socioeconom*[tiab] OR economic*[tiab]** | **4054185** |
| **3. Perception** | **“Bias”[Mesh] OR** “Body Image”[Mesh] OR “Beauty”[Mesh] OR beauty[tiab] OR beautif*[tiab] OR attract*[tiab] OR “body image”[tiab] OR bias[tiab] OR appearance[tiab] OR appearances[tiab] OR percept*[tiab] OR perceiv*[tiab] OR advantag*[tiab] OR disadvantag*[tiab] OR satisf*[tiab] OR “self image”[tiab] OR “self-image”[tiab] OR “self esteem”[tiab] OR “self-esteem”[tiab] OR “self confidence”[tiab] OR “self-confidence”[tiab] | **2661989** |
| **4. COMBINE** | **#1 AND #2 AND #3** | **1019** |
|  |  |  |
|  |  |  |
|  |  |  |
|  |  |  |
| **Validation String** | **34054661 OR 30505018 OR 28448667 OR 39357139 OR 9220717 OR 15529199 OR 25991993 OR 28301645 OR 30520214 OR 30252808 OR 31219525 OR 31294743 OR 33123780 OR 37731729 OR 25909302 OR 28571061 OR 31832733 OR 35169915 OR 38442513 OR 39694907 OR 26008224 OR 26579860 OR 30543344 OR 35501055 OR 37497162 OR 39155133 OR 26063837 OR 29135891 OR 33374702 OR 34033632 OR 38347975 OR 38864039 OR 39130903 OR 39952155 OR 39991361 OR 6613742 OR 15959689 OR 17551776 OR 25270095 OR 27140047 OR 26818280 OR 32366125 OR 36268461 OR 38196074 OR 38331035 OR 38336001 OR 38881698 OR 39441951** | **12/48** |

**(("Rhinoplasty"[Mesh] OR "Blepharoplasty"[Mesh] OR "Rhytidoplasty"[Mesh] OR ((cosmetic[tiab] OR aesthetic[tiab] OR facial[tiab]) AND (surger*[tiab] OR procedur*[tiab] OR operati*[tiab])) OR facelift[tiab] OR facelifts[tiab] OR "jawline contouring"[tiab] OR rhinoplasty[tiab] OR rhinoplasties[tiab] OR blepharoplasty[tiab] OR blepharoplasties[tiab] OR Botox[tiab] OR ((face[tiab] OR facial[tiab] OR lip[tiab] OR lips[tiab] OR cheek[tiab] OR cheeks[tiab]) AND (inject*[tiab] OR filler[tiab] OR fillers[tiab])) OR rhytidoplasty[tiab] OR rhytidoplasties[tiab] OR "face lift"[tiab] OR "face lifts"[tiab] OR septorhinoplast*[tiab]) AND ("Employment"[Mesh] OR "Job Application"[Mesh] OR "Personnel Selection"[mesh] OR career*[tiab] OR workplace*[tiab] OR job[tiab] OR jobs[tiab] OR hired[tiab] OR hiring[tiab] OR hire[tiab] OR hires[tiab] OR employ*[tiab] OR unemploy*[tiab] OR work*[tiab] OR interview*[tiab] OR socioeconom*[tiab] OR economic*[tiab])) AND ("Bias"[Mesh] OR "Body Image"[Mesh] OR "Beauty"[Mesh] OR beauty[tiab] OR beautif*[tiab] OR attract*[tiab] OR "body image"[tiab] OR bias[tiab] OR appearance[tiab] OR appearances[tiab] OR percept*[tiab] OR perceiv*[tiab] OR advantag*[tiab] OR disadvantag*[tiab] OR satisf*[tiab] OR "self image"[tiab] OR "self-image"[tiab] OR "self esteem"[tiab] OR "self-esteem"[tiab] OR "self confidence"[tiab] OR "self-confidence"[tiab])**
