# Supplementary material for: From Nose Job to a Better Job: A Scoping Review of Facial Aesthetics, Attractiveness Bias, and Outcomes in the Workplace
Source: Aesthet Surg J Open Forum. 2026 Jun 8;8:ojag104. doi: 10.1093/asjof/ojag104 (PMC13322391; doi:10.1093/asjof/ojag104)
Supplement: ojag104_Supplementary_Data [file ojag104_supplementary_data.zip › Supplememtary Table 1.docx]

| **Study** | **Year** | **Study Type** | **Sample Size** | **Population Characteristics** | **Outcome Measures** | **Key Findings** | **Category** |
| --- | --- | --- | --- | --- | --- | --- | --- |
| Abousy et al. | 2022 | cross sectional | 815 | Laypersons crowdsourced to complete survey | external perception after facial transplantation | post-transplant males and females were perceived globally as more professionally competent and socially accepted | External Perception and Attractiveness Bias |
| Baptista et al. | 2025 | randomized clinical trial | 30 | women between age 45-65 years who underwent rhytidectomy | change perceived age, judged by human and AI raters | All groups who received different techniques for rhytidectomy (deep plane, high SMAS, etc) resulted in decreased age (-0.80 — -2.40 years, based on technique; no statistical signifant difference between techniques) in both human and AI judgement of age. | External Perception and Attractiveness Bias |
| Bater et al. | 2017 | cross sectional | 504 | adult laypersons compleeting a Qualtrics survey | perception of age of photographs pre-/ and post-facial rejuvenation surgery | post-surgical patients rated as younger (-4.61 years), more attractive, successful, and healthy. | External Perception and Attractiveness Bias |
| Bater et al. | 2018 | cross sectional | 401 | adult laypersons compleeting a Qualtrics survey | perception of age of photographs pre-/ and post-blepharoplasty | Post-surgical patients rated as younger (-1.04 years), more attractive, healthy, and having a higher energy level | External Perception and Attractiveness Bias |
| Broer et al. | 2012 | cross sectional | 1226 | American adults (720 of which were plastic surgeons) | comparison of ideal nose shape between different cultural identities | Measurements for ideal noses across cultures were very similar across general populations across the globe. | External Perception and Attractiveness Bias |
| Broer et al. | 2014 | cross sectional | 1226 | American adults (720 of which were plastic surgeons) | comparison of ideal facial characteristics between different cultural identities | ideal measurements for lip and chin projection differed based on ethnic background | External Perception and Attractiveness Bias |
| Cassidy et al. | 2023 | cross sectional | 249 | 119 undergraduate students at Indiana University; 127 undergraduate students at Boğaziçi University | pleasantness scores measured directly and indirectly based on facial attractiveness across two separate cultures | increased facial attractiveness is associated with increased pleasantness scores, directly and indirectly, though directly had a larger effect | External Perception and Attractiveness Bias |
| Frautschi et al. | 2020 | prospective cohort | 25 | adults (22-59 years old) composed of laypersons and individuals with varied medical training | perceived age of photographs pre- and post-rhytidectomy and tracking eye patterns. | all patients (female patients aged 55-71) experienced reduction in apparent age (5.26 ± 3.3 years) post-operatively. Less visual attention given to neck, upper third, and perioral region post- vs. pre-operatively. | External Perception and Attractiveness Bias |
| Ishii et al. | 2016 | prospective cohort | 60 | layperson adult persons | measurement of eye-tracking in petients with facial paralysis vs patients without paralysis | extra attention was paid to the mouth of the paralyzed patient, especially during smiling, with a preference for the nonparalyzed side. Smiling induced a significant gaze deviation to the nonparalyzed side of the mouth. | External Perception and Attractiveness Bias |
| Kalick et al. | 1979 | cross sectional | 96 | Boston-area college students | external perception of personality traits before and after rhinoplasty, mentoplasty, or both | patients were perceived as more attractive, socially likeable, having higher "marital competence," and more successful; this effect was greater in males evaluating females versus females evaluating females | External Perception and Attractiveness Bias |
| Kandathil et al. | 2018 | cross sectional | 813 | English-speakering adults | external perception of personality traits before and after rhinoplasty | photos post-surgery are perceived as more approachable, attractive, and healthy | External Perception and Attractiveness Bias |
| Liang et al. | 2024 | cross sectional | 446 | Adult Han Chinese men recruited online | attractiveness, loyalty ratings, and mating contexts of photos that have received cosmetic surgery, have make up, or no make up. | Patients post-cosmetic facial surgery were perceived as more attractive when participants were unaware of the surgery, | External Perception and Attractiveness Bias |
| Masnari et al. | 2012 | cross sectional | 87 | pediatric patients (9 months-16 years) or their family members (if <7 years old) with either facial burn scar > 6 months post-injury, hemangioma, port-wine stain, or congenital melanocytic nevus | perceived stigmatization, socioeconomic status, and mental health of the parents | children with facial differences are at high risk for experiencing stigmatising behaviours, such as staring, startled reactions, teasing and expressions of pity | External Perception and Attractiveness Bias |
| Mazzaferro et al. | 2017 | cross sectional | 476 | adults who were Mechanical Turk respondents with a minimum 95 percent approval rating and were living in the United States | perceived personality traits and emotional expressions pre- and post-orthognathic surgery | post-operative photos were perceived as more dominant, trustworthy, friendly,  intelligent, attractive, and less threatening (effect for dominance more pronounced by participants with income >$100,000/year). Also perceived to be happier and less angry, surprised, sad, afraid, and disgusted. | External Perception and Attractiveness Bias |
| Necka et al. | 2021 | cross sectional | 1108 | adults who were Mechanical Turk respondents with at least 90% approval rating residing in the United States | expected perceived pain based solely on physical appearance of simulated clinician doing painful procedure | participants expected to experience less pain and be more likely to use over-the-counter medication versus prescription pain medication if treated by the "more competent" looking provider. Same effect for similarities between rater and provider | External Perception and Attractiveness Bias |
| Rankin et al. | 2003 | cross sectional | 210 | Adults that were patients of a hospital within a three state area in the United States | perceptions of those with facial deformities | patients with facial deformities were assessed as less honest, trustworthy, attractive, intelligent, effective, popular, optimistic, and capable | External Perception and Attractiveness Bias |
| Roxbury et al. | 2012 | cross sectional | 39 | Adult persons entering the main entrance of the Johns Hopkins Outpatient Center | asymmetry of patients' noses pre- and post-rhinoplasty, then attractivness ratings of said patients | surgical straightening of a nose resulted in an 8% improvement in attractiveness | External Perception and Attractiveness Bias |
| Sinko et al. | 2018 | cross sectional | 155 | Adult raters (19-42 years old) from predominantly Austria | perceived personality traits before and after surgery | orthognathic patients post-surgery were rated as more beautiful, successful, pleasant, faithful, attractive, intelligent, good-natured, healthy, erotic, and confident, with class III patients having generally the largest change after surgery | External Perception and Attractiveness Bias |
| Vuyk et al. | 1995 | retrospective observational | 15 | Patients selected from a Dutch private practice | social perception of patients by others | Six of 13 rhinoplasty patients were judged more positive after the operation. Effects of the operation did not depend on the gender of the patients. 7 of the 15 patients were judged more positive postoperative than preoperative. | External Perception and Attractiveness Bias |
| Şimşek et al. | 2024 | retrospective cohort | 579 | Adult patients (18-65 years old) at a single plastic surgery practice | prevalence of body dysmorphic disorder | 8.6% (27/313) of patients seeking cosmetic procedures had body dysmorphic disorder | Mental Health and Appearance-Related Distress |
| Adamidou et al. | 2024 | interpretive phenomenological analysis | 6 | Greek women >18 years old | causes of negative body image | Struggling to fit in to society expectations of beauty has led to use of cosmetic surgery to enhance personal confidence, or the desire for surgery for the same reason | Mental Health and Appearance-Related Distress |
| Dinis et al. | 1998 | cross sectional | 25 | adult patients referred to ENT outpatient clinic for septorhinoplasty | evaluate patient's state of mind pre- and post-operatively | Pre-op: 40% (10/25) patients had some "psychiatric disturbance": 5 had a personality disorder, 6 had a depressive-type adjustment (1 patient included in both). Post-surgical 3/6 depressive-type adjustment disorder patients showed improvement in depressive symptoms. | Mental Health and Appearance-Related Distress |
| Dowling et al. | 2010 | prospective, quantitative cohort | 50 | Australian adult male cosmetic surgery patients | compare presurgical motivations and expectations and postsurgical satisfaction between male and female cosmetic surgery patients | More males than females (44% vs 30%) reported being bullied because of their appearance and their appearance affected their sexual relationships (32% vs 17%) presurgery. Most common motivation was to improve self-image. No evidence of increased psychosocial dysfunction between sexes. Men less satisfied with results postsurgery (81% vs 94%) | Mental Health and Appearance-Related Distress |
| Gulbas | 2013 | ethnographic qualitative study | 499 | adult women in Venezuela | attitudes toward participants' noses, desire for cosmetic surgery, and lived experiences because of their appearance | most frequently desired body part to have altered was the nose, (16% "blanca" patients, 16% "morena," and "nearly 50% Afro-Venezuelan." Wider broader noses led to perceived racial discrimination | Mental Health and Appearance-Related Distress |
| Ip et al. | 2019 | mixed-methods study | 3 | young adult women who experienced childhood psychological trauma and have received cosmetic surgery in adulthood | participants attitudinal dispositions toward the physical self, the fear of negative feedback on their appearance, and the acceptance of cosmetic surgery, respectively. | all participants believed that cosmetic surgery enhanced their confidence and body image | Mental Health and Appearance-Related Distress |
| Jalali et al. | 2021 | qualitative grounded theory study | 20 | 16 participants, two nurses, one rhinoplasty surgeon, and one participant's family member in Iran | motivation to pursue rhinoplasty | Physical attractiveness affected all aspects of participants' lives due to being unhappy with the appearance of their nose. This affected their mood and took away their self-confidence to the point that they considered themselves as being inferior to other more attractive people... Some participants believed that they were treated differently by people in society based on their appearance. | Mental Health and Appearance-Related Distress |
| Joseph et al. | 2017 | prospective cohort | 84 | preoperative adult patients seeking rhinoplasty | prevalance of body dysmorphic disorder in rhinoplasty patients | higher rates of high-risk for body dysmorphic disorder versus control (32% vs 4%) | Mental Health and Appearance-Related Distress |
| Mironica et al. | 2024 | systematic review | 25 | English language articles from 2013-2023 identified by "Mesh term "aesthetic surgery" and the keywords "cosmetic surgery," "social media," and "body image dissatisfaction."" | factors associated with likeliness to pursue cosmetic surgery and attitudes towards it--positive and negative | among positive attitudes, enhances appearance and improved emotional well-being are reasons. One factor associated with likeliness to pursue is body dissatisfaction and anxiety | Mental Health and Appearance-Related Distress |
| Pearl et al. | 2019 | cross sectional | 50 | Patients from a university-based, single-surgeon, aesthetic plastic surgery clinic (University of Pennsylvania) | reasons for experiences discrimination and psychological outcomes related to said discrimination | Age was main reason for experienced discrimination (31.9%), in following settings: interpersonal context (36.0%), work (20.0%), and romantic(16.0%) or healthcare (16.0%). Associated with poorer rated health and lower self-esteem | Mental Health and Appearance-Related Distress |
| Qian et al. | 2021 | cross sectional | 426 | Adult patients seeking "cosmetic treatment" (i.e., surgical and nonsurgical) in China | correlating personality traits with desire for cosmetic treatments, including which traits are associated with which procedures | Those with higher rates of neuroticsim had higher treatment tendency for rhinoplasty (OR 1.07), blepharoplasty (OR 1.04) and body (OR 1.06). Higher rates of psychoticism also associated with higher rates of cosmetic eye surgery (OR 1.10) | Mental Health and Appearance-Related Distress |
| Shandilya et al. | 2024 | retrospective cohort | 1602 | Adult rhinoplasty patients at one surgical clinic in Ireland | incidence of body dysmorphic disorder | 2.5% (22/892) diagnosed by psychiatrist having mild body dysmorphic disorder; 15 of these patients were operated on successfully | Mental Health and Appearance-Related Distress |
| Stepp et al. | 2023 | cross sectional | 29 | Adult patients recruited from medical and dental providers at the Unviersity of North Carolina | prevalance of facial body dysmorphic disorder in adult patients living with cleft lip and palate | 93.1% (n=23) patients reported complications in social relationships due to experiences with their appearance and negative self-image. More likely to screen positive for body dysmorphic disorder and more likely to have higher scores | Mental Health and Appearance-Related Distress |
| Alduosari et al. | 2025 | cross sectional | 730 | Kuwaiti nationals >18 years old | liklihood of undergoing cosmetic surgery based on social media usage | those who regularly engage in social media--particularly in young adults--are more likely to undergo cosmetic surgery to align with Western beauty standards | Self-Perception and Psychosocial Outcome |
| Almajnoni et al. | 2023 | cross sectional | 1249 | general adult population from the western province of Saudi Arabia | overall acceptance of plastic surgery | over half (54.2%) of respondents in support of plastic surgery. Most common cosmetic surgery received was rhinoplasty (15.8%) | Self-Perception and Psychosocial Outcome |
| Alsulaiman et al. | 2023 | cross sectional | 135 | female high school students in Riyadh City, Saudi Arabia | interest in rhinoplasty | over half (51.9%) of 17 year olds were interested in a rhinoplasty; most common reason was to "enhance beatuy;" most common desire among those who had a family member who had had a rhinoplasty in the past | Self-Perception and Psychosocial Outcome |
| Amantayeva et al. | 2024 | cross sectional | 515 | Kazakh citizens | 1. attitudes towards plastic surgery and plans to undergo plastic surgery 2. reasons for undergoing cosmetic plastic surgery and most common procedures in Kazakhstan | 40% participants express desire to undergo cosmetic surgery. 1 in 6 citizens in largest city (Almaty) is or has been patient in cosmetic clinic. Most common procedure most popular plastic surgery in Almaty is "blepharoplasty with Europeanization of the eyelids." | Self-Perception and Psychosocial Outcome |
| Arkoubi et al. | 2024 | cross sectional | 3238 | Adults in Saudi Arabia (>18 / <60 years old) | quantify prevalence and determinants of plastic surgery amongst citizen of Saudi Arabia | 1328 (41%) participants had undergone plastic surgery. Most common procedure (31.2%) was breast augmentation; 3.5% received facial cosmetic surgery. Most common reason to enhance self-image (1394; 47.3%); 1206 (37.2%) desired to be "more socially accepted, make friends, find a partner, or secure employment." | Self-Perception and Psychosocial Outcome |
| Balaji et al. | 2019 | cross sectional | 23 | Males seeking facial cosmetic surgery in India | qualitatively define expectations from male patients undergoing facial cosmetic surgery | most common inquiries related to midface-specifically nose, zygoma, or maxilla. Nose to fit within the global, Westernized perception of midface esthetics. Desire for surgery because they desire personality trait associated with certain appearance (i.e., aggression, increase in “winning tendency” or “higher in corporate or social hierarchy.”) | Self-Perception and Psychosocial Outcome |
| Baniulyte et al. | 2024 | prospective, observational cohort | 64 | orthognathic surgery patients from one surgeon's practice | reasons for and benefits of surgery | most commonly chosen reasons were "improved facial appearance" (84% pre-surgery/90% post-surgery)), "improved dental appearance" (80%/95%), and "happy to have photos/video taken" (77%/80%), then "improved eating" (69%/75%) (note, "improved eating" was the most commonly chosen main reason for undergoing surgery pre-surgery [24%], but "improved facial appearance" was the main benefit post surgery [21%]) | Self-Perception and Psychosocial Outcome |
| Borujeni et al. | 2020 | quasi-experimental study | 100 | rhinoplasty patients admitted to one surgical clinic. | factors related to self-esteem | Body image and self-esteem improved significantly after surgery. | Self-Perception and Psychosocial Outcome |
| Cingi and Eskiizmir | 2013 | prospective cohort | 81 | 191 rhinoplasty patients | patient satisfaction and quality of life + angles of deviations of deviated noses | improved satisfaction with appearance, family/friends' satisfaction with appearance, social interactions, and self-confience across groups. Non-deviated groups had larger increases than deviated group in all except friends' satisfaction with appearance (*p* <0.012 across domains) | Self-Perception and Psychosocial Outcome |
| Du et al. | 2025 | prospective cohort | 171 | adult patients receiving reduction mandibuloplasty | FACE-Q outcome measures | psychological well-being (75.93 vs 49.44) and social confidence (71.23 vs 58.28) increased post-surgery. | Self-Perception and Psychosocial Outcome |
| Huynh et al. | 2020 | mixed-methods study | 315 | adult female patients of east or southeast Asian descent that either had expressed or had undergone blepharoplasty | attitudes and motivations for an Asian blepharoplasty | 6 independent factors for Asian blepharoplasty: one's understanding of how one is perceived by others (Looking Glass Self index), a desire to advance socially and professionally (Desire for Social-Professional Advancement index), functional impairment, encouragement from family or friends, an appearance of fatigue, and asymmetry of the eyelid. | Self-Perception and Psychosocial Outcome |
| Kaewsomnuck et al. | 2024 | cross sectional | 1452 | Adults in Thailand (18-60 years old) | motivations for rhinoplasty and ideal nasal profiles | primary motivations were "aethetic enhancement" (76.69%), "increased self-confidence" (72.25%), and "help in occupation" (28.52%). Ideal nasal profile slightly wider alar-intercanthal distance than established western literature (1.05 for females/1.10 for males vs 1.00) | Self-Perception and Psychosocial Outcome |
| Kasnakaglu | 2021 | cross sectional | 31 | Adult female patients pursuing "anti-aging" operations at one hospital (Gazi University Hospital, Ankara, Turkey | different perceptions of aging and how that affects desire for cosmetic surgery | viewed as individual responsibility to "maintain youth and look good," and surgical procedures are "things to buy" in the market similar to other beautification tools. Driving factors were desire to regain old self, enhance current self, or to desire new self | Self-Perception and Psychosocial Outcome |
| Kinnunen | 2010 | qualitative phenomenological-hermeneutic study | 36 | Finnish adults (ages 55-84 years old) who had undergone plastic surgery and (n=23), Finnish plastic surgeons or consultants (n=14) | motivations to pursue cosmetic surgery | themes that emerged included "well-deserved compensation" for rapid aging due to difficult life, "regaining respectability," and "curing the melancholic Finnish body." | Self-Perception and Psychosocial Outcome |
| Levine et al. | 2005 | retroactive case control | 20 | Adult patients (18-45 years old) with either a laceration of 3 cm or greater and/or a fractured facial bone requiring operative intervention | personal and external perceptions after facial trauma | Participants had statistically significant lower satisfaction with life and higher rates of unemployment post-trauma and were perceived as statistically significantly less attractive by others | Self-Perception and Psychosocial Outcome |
| Locatelli et al. | 2017 | prospective qualitative cohort | 35 | 30 female and 5 male cosmetic surgery patients at Hopital Saint-Louis, Paris, France | motivation to pursue aesthetic surgery | Driving factors related to recent events leading to or strengthening a desire to change appearance based on broad psychological needs, to increase self-image | Self-Perception and Psychosocial Outcome |
| Öztürk et al. | 2020 | cross sectional | 100 | Adult rhinoplasty patients at one surgical clinic in Turkey | comparing the self-esteem, body perception, and social media use of individuals with and without a history of rhinoplasty | higher social media addiction scores among rhinoplasty patients versus control, but no difference in meeting diagnostic threshold. No difference in self esteem nor body perception scores | Self-Perception and Psychosocial Outcome |
| Papadopulos et al. | 2019 | retroactive case control | 46 | adult patients (38-75 years old) who underwent transdermal blepharoplasty between 1995-2008 at one institution (University Hospital rechts de Isar, Munich, Germany) for solely cosmetic reasons | general life satisfaction, satisfaction with health, and subjective evaluation of the procedure | increased life satisfaction in domains of "leisure time/hobbies" and "work." Increased self-esteem and emotionality and self-assessment | Self-Perception and Psychosocial Outcome |
| Patil et al. | 2011 | cross sectional | 872 | umarried women (18-30 years old) residing in rural villages in India | source of information, awareness of, and willingness to undergo aesthetic surgery | most common region of aesthetic surgery recognized as facial aesthetics (35.7%). 85.7% (711) patients felt they required aesthetic surgery, with most common area being the face (40.9%) | Self-Perception and Psychosocial Outcome |
| Rabah et al. | 2025 | cross sectional | 726 | Adults residing in Saudi Arabia | perceptions, attitudes, and motivations surrounding rhytidectomy | most commonly cited motive for undergoing facelift was increased self-confidence (39%). | Self-Perception and Psychosocial Outcome |
| Robin et al. | 1988 | prospective cohort | 31 | adult patients undergoing rhinoplasty | self-perceived attractiveness and mental state post-rhinoplasty | both self-perceived appearance and mental state improved post-proceudre | Self-Perception and Psychosocial Outcome |
| Rodgers et al. | 2024 | cross sectional | 308 | English-speaking adult women recruited online | cosmetic procedures or projects received and pressures for doing so | media pressure was strongest influencer. 11.2% of women had received facial cosmetic surgery, but another 38% were considering it | Self-Perception and Psychosocial Outcome |
| Salehahmadi et al. | 2012 | cross sectional | 101 | Adult patients (81 female/20 male) seeking cosmetic surgery at one practice in southern Iran | influencial factors driving patients to cosmetic surgery | self-improvement, finding a better job opportunity, rivalry, media, health status | Self-Perception and Psychosocial Outcome |
| Timraz et al. | 2024 | systematic review | 8 | Adult Saudi cosmetic surgery patients | Articles discussing the role of social media influence on cosmetic surgery decisions among adult Saudi patients | increased social media exposure leads to higher body dissatisfaction and lower self-esteem. More exposure leads to higher likelihood to pursue cosmetic surgery, especially in 20-30 age bracket. | Self-Perception and Psychosocial Outcome |
| Vasović et al. | 2024 | prospective randomized controlled trial | 348 | adult patients (49-87 years old) who underwent upper blepharoplasty in Helsinki | FACE-Q outcome measures at 3, 6, and 12 months post-operatively | early-life impact significanly increased with each follow uo (40;66;73;77, *p* < 0.001). Improvements across satisfaction with eyes, face, and overall outcome were improved at different phases, and both social and psychological function were improved by the 3 month follow-up (40;77, p < 0.001) (41;66 p < 0.001) | Self-Perception and Psychosocial Outcome |
| Viana et al. | 2011 | prospective randomized controlled trial | 50 | Adult patients recruited from Ophthalmic Plastic Surgery Clinic seeking lower blepharoplasty | Rosenberg Self-Esteem scale measures | 62% (31/50) patients had improved self-esteem after surgery, 24% (12/50) had unchanged scores, 14% (7/50) had worsened self-esteem. However, 100% of patients reported positive changes in their social lives and relationships. Average reduction of 1.5 on RSES-EPM in post-op score | Self-Perception and Psychosocial Outcome |
| Wu et al. | 2022 | cross sectional | 40 | Adult females (18-50 years old) in the Netherlands and in China | attitudes and motivations for cosmetic surgery across two different cultures | Beauty described as capitalizable by all Chinese women and two Dutch women. All Chinese women and 12 Dutch women described being more attractive to wealthy men. Other sociocultural themes were not as globally recognized, though still acknowledged across cultures | Self-Perception and Psychosocial Outcome |
| Yin et al. | 2016 | prospective, observational cohort | 163 | Female patients (18-30 years old) from the Plastic Surgery Hospital, Chinese Academy of Medical Sciences | evaluation of self-esteem and self-efficacy, subjective and objective assessment of facial appearance, and structural equation models | Mild to moderate impairments to self-esteem and self-efficacy identified in pre-operative patients versus controlled, nonsurgical population, followed by increase in self-esteem (22.60;25.88. *p*<0.001) and self-efficacy (21.50;26.38, p<0.001) | Self-Perception and Psychosocial Outcome |
| Zamani and Fazilatpour | 2013 | quasi-experimental study | 45 | convenience sampling from a waiting list of rhinoplasty in one cosmetic surgery clinic, waiting list for a general operation, or who were not waiting for any operation | evaluation of self-esteem and body image before operation, one week after, and 2 months after | Significant improvements in self-esteem (F=24.54, *p*<0.001) and body image (F=23.21 *p*<0.001) post-operatively were only seen in the cosmetic surgery group. | Self-Perception and Psychosocial Outcome |

**Supplementary Table 1.** Characteristics of Included Studies. Summary of 61 studies included in this scoping review, detailing study design, sample size, population characteristics (including geographic distribution when reported), outcome measures, key findings, and to which conceptual domain each study was assigned.
